# Osteoarticular Infections Caused by Non-*Aspergillus* Filamentous Fungi in Adult and Pediatric Patients: A Systematic Review

**DOI:** 10.1097/MD.0000000000002078

**Published:** 2015-12-18

**Authors:** Saad J. Taj-Aldeen, Blandine Rammaert, Maria Gamaletsou, Nikolaos V. Sipsas, Valerie Zeller, Emmanuel Roilides, Dimitrios P. Kontoyiannis, Andy O. Miller, Vidmantas Petraitis, Thomas J. Walsh, Olivier Lortholary

**Affiliations:** From the Mycology Unit, Microbiology Division, Department of Laboratory Medicine and Pathology, Hamad Medical Corporation, Doha, Qatar (SJT-A); Center for Osteoarticular Mycoses, Hospital for Special Surgery (SJT-A, BR, MG, NVS, ER, AOM, VP, TJW, OL); International Osteoarticular Mycoses Study Consortium, NY (SJT-A, BR, MG, NVS, ER, AOM, VP, TJW, OL); Weill Cornell Medical College, Doha, Qatar (SJT-A); Université Paris-Descartes, Sorbonne Paris Cité, APHP, Service des Maladies Infectieuses et Tropicales, Hôpital Necker-Enfants Malades, Centre d’Infectiologie Necker-Pasteur, Institut Imagine (BR, OL); Institut Pasteur, Mycology Molecular Unit, Paris, France (BR, OL); Transplantation-Oncology Infectious Diseases Program, Department of Medicine, Weill Cornell Medical Center of Cornell University (MG, AOM, VP, TJW); Pediatrics, and Microbiology & Immunology, Weill Cornell Medical Center of Cornell University, New York, NY (MG, NVS, TJW); National and Kapodistrian University of Athens, Athens, Greece (MG, NVS); Osteoarticular Reference Center, Groupe Hospitalier Diaconesses-Croix Saint-Simon, Paris, France (VZ); Infectious Diseases Unit, ^3rd^ Department of Pediatrics, Faculty of Medicine, Aristotle University, School of Health Sciences, and Hippokration Hospital, Thessaloniki, Greece (ER); and MD Anderson Cancer Center, Houston, TX (DPK).

## Abstract

Osteoarticular mycoses due to non-*Aspergillus* moulds are uncommon and challenging infections.

A systematic literature review of non-*Aspergillus* osteoarticular mycoses was performed using PUBMED and EMBASE databases from 1970 to 2013.

Among 145 patients were 111 adults (median age 48.5 [16–92 y]) and 34 pediatric patients (median age 7.5 [3–15 y]); 114 (79.7%) were male and 88 (61.9%) were immunocompromised. Osteomyelitis was due to direct inoculation in 54.5%. Trauma and puncture wounds were more frequent in children (73.5% vs 43.5%; *P* = 0.001). Prior surgery was more frequent in adults (27.7% vs 5.9%; *P* = 0.025). Vertebral (23.2%) and craniofacial osteomyelitis (13.1%) with neurological deficits predominated in adults. Lower limb osteomyelitis (47.7%) and knee arthritis (67.8%) were predominantly seen in children. Hyalohyphomycosis represented 64.8% of documented infections with *Scedosporium apiospermum* (33.1%) and *Lomentospora prolificans* (15.8%) as the most common causes. Combined antifungal therapy and surgery was used in 69% of cases with overall response in 85.8%. Median duration of therapy was 115 days (range 5–730). When voriconazole was used as single agent for treatment of hyalohyphomycosis and phaeohyphomycosis, an overall response rate was achieved in 94.1% of cases.

Non-*Aspergillus* osteoarticular mycoses occur most frequently in children after injury and in adults after surgery. Accurate early diagnosis and long-course therapy (median 6 mo) with a combined medical-surgical approach may result in favorable outcome.

## INTRODUCTION

Fungal osteomyelitis and arthritis are uncommon diseases that generally present in an indolent manner. Being one of the most challenging complications in orthopedic and trauma surgery, fungal osteoarticular infections often require complex treatments in specialized centers. The majority of these infections are caused by *Aspergillus*^1–3^ and *Candida* species.^4,5^ Other osteoarticular infections are reported with dimorphic fungi and *Cryptococcus neoformans,* which demonstrate distinctive clinical presentations, occur predominantly in immunocompetent patients, and develop from hematogenous dissemination.^6^

Opportunistic infections due to other groups of fungi are increasingly reported as potential emerging pathogens, but with limited description and relatively few reports of osteoarticular mycoses. Although a management algorithm was proposed recently for such fungal bone and joint infections,^7^ no comprehensive literature analysis addresses the demographic, clinical aspects, microbiology, therapy, and outcome of osteoarticular infections caused by non-*Aspergillus* moulds. The possible mechanisms of infection that cause osteomyelitis or arthritis are also not well documented. The portal of entry and the ability to disseminate may differ for each group of fungi. Furthermore, many clinical, diagnostic, and therapeutic questions remain uncertain.

We therefore conducted an extensive literature review to study bone and joint infections by hyaline hyphomycetes, Mucorales, and dematiaceous moulds. Using highly detailed case criteria of host factors, symptoms, physical findings, disease features, diagnostic imaging, management, and outcome, we compiled the characteristic clinical manifestations and treatment modalities of these serious invasive fungal diseases.

## METHODS

### Search Criteria

To identify fungal osteomyelitis and arthritis caused by hyaline hyphomycetes, Mucorales, and dematiaceous fungi, we used the OvidSP search platform in the MEDLINE and EMBASE databases using the following keywords: fungi, Ascomycota, Pseudallescheria, Chaetomium, Schizophyllum, Mucorales, mitosporic fungi, Acremonium, Alternaria, Beauveria, Chrysosporium, Cladosporium, Exophiala, Fusarium, Helminthosporium, Madurella, Phialophora, Scedosporium, Scopulariopsis, Trichoderma, Ascomycetes, Basidiomycetes, blastocladiomycota, Deuteromycetes, zygomycetes, zygomycosis, systemic mycosis, entomophthoromycosis, mucormycosis, bone diseases, bone infection, osteitis, osteomyelitis, periostitis, spondylitis, discitis, osteochondritis, osteomyelitis, periostitis, infectious arthritis, bone and joint infections, and reactive arthritis. Qatar Foundation proposal number NPRP 5-298-3-086 approved the study; the ethical approval was not necessary for the retrospective literature review nature of the research.

**FIGURE 1:**
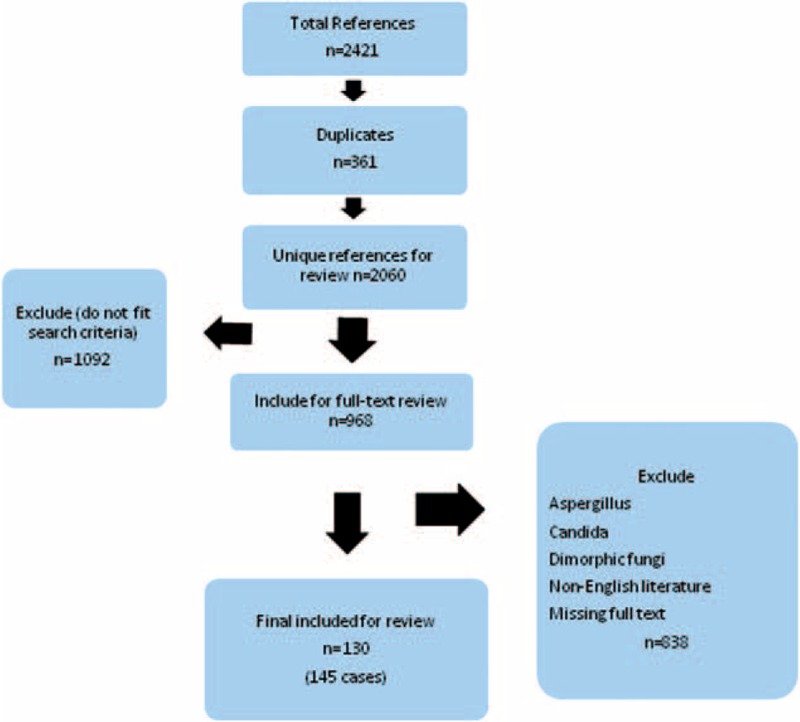
Flow diagram of search and included studies.

We retrieved a total of 2421 references published from January 1970 to September 2013. Figure 1 shows the selection process applied to identify the osteoarticular infections.

We included cases in the final analysis with data on osteomyelitis and or arthritis, site of infection, underlying disease, and medical and surgical therapy. Other parameters also considered in case analysis were radiological images, inflammatory markers, and disease manifestations. We excluded cases with bone extension from rhinosinusitis, outbreak of *Exserohilum rostratum* infections after injection of contaminated glucocorticosteroids in USA,^8^ as well as cases with missing full texts, and cases of non-English literature.

### Data Extraction

The following parameters were extracted from each study when present: age, sex, risk factors, prior surgery, treatment, antifungal agent, duration of treatment, time to diagnosis, fever, inflammatory markers, neutropenia, radiological features, type of bone infection, surgical intervention, histopathology, microscopy, culture, fungal species, and outcome.

### Synonyms of Fungi

Due to taxonomic changes since the year 1970,^9–12^ fungi in this study are referred with their current name: *Scedosporium boydii* (formerly *Pseudallescheria boydii*, or *Petriellidium boydii*, or *Allescheria boydii*), *Scedosporium apiospermum* (formerly *Monosporium apiospermum*, or *Pseudoallescheria apiosperma*), *Lomentospora prolificans* (formerly *Scedosporium prolificans*, or *Scedosporium inflatum*), *Lichtheimia corymbifera* (formerly *Absidia corymbifera* and *Mycocladus corymbifera*), and *Fusarium falciforme* (formerly *Acremonium falciforme*).

### Definitions

All definitions used throughout this study were referred to the previously published definitions.^1–5^ The following definitions were related to the mechanism of bone infection, criteria for diagnostic probability, onset of infection, and therapeutic response.

1. Direct inoculation: Local bone or joint infection after a skin breakdown.
2. Hematogenous: Seeding to bone or joint by dissemination from a distant site of inoculation/infection.
3. Contiguous: Seeding to bone or joint from an adjacent infection site.
4. Proven fungal osteomyelitis: Evidence of a positive culture, and/or histology from bone tissue, joint fluid, or metal hardware.
5. Probable fungal osteomyelitis: Compatible clinical and radiological features of osteomyelitis with evidence of positive histology and/or fungal culture from an extraosteoarticular site.
6. De novo fungal osteomyelitis: Clinically apparent onset of fungal osteomyelitis in a patient not concomitantly receiving antifungal agents for an invasive fungal disease.
7. Overall response: Complete or partial resolution of clinical and radiological findings of osteomyelitis.
8. Children definition: Patients ≤15 years.
9. C-reactive protein (CRP) was elevated when >1 mg/dL; white blood cell (WBC) count was elevated when >10,000/mm^3^

### Data Analysis and Statistical Methods

Descriptive statistics were used to summarize all demographic and clinical characteristics of the patients. Quality of data (review of completeness, data verification, and validation and accuracy of data) was assessed by the lead investigators. Quantitative variables are presented as mean ± standard deviation (SD) or as medians (with range). Differences between continuous variables of at least 2 independent groups were analyzed using unpaired *t* test and 1-way analysis of variance (ANOVA). When an overall group difference was found to be statistically significant, pairwise comparisons were made using the appropriate post-hoc test. Differences between categorical variables were analysed using chi-square test and Fisher exact test, as appropriate. Relationships between 2 continuous variables were further examined using Pearson correlation coefficients. Pictorial presentations of the key results were made using appropriate statistical graphs. All *P* values presented were 2-tailed, and *P* values <0.05 were considered as statistically significant. All statistical analyses were done using statistical packages SPSS 19.0 (SPSS Inc. Chicago, IL).

## RESULTS

### Population, Demographic Characteristics, and Comorbidities

**FIGURE 2:**
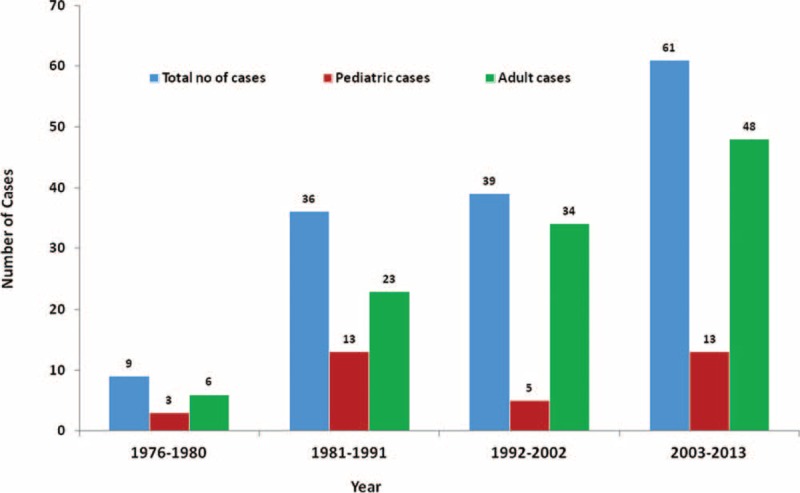
Number of osteoarticular infections caused by non-*Aspergillus* fungal species reported in the literature from 1970 to 2013.

A total of 145 individual cases from 130 publications^13–142^ of osteoarticular infections fulfilled the definition criteria. Cases were classified as proven in 51% (n = 74) and probable in 49% (n = 71). The number of reports increased over time particularly in the adult population (Fig. 2). The demographic characteristics of the 145 cases are described in Table 1. Among the 111 adults and 34 children, male patients predominated (79.7%). The underlying conditions identified for the majority of patients included trauma or puncture wound (51.4%), prior surgery (22.5%), and diabetes (15.5%). Corticosteroid use was reported in 16.2% of the cases. Severe immunocompromised patients including solid cancer, hematological malignancy, transplantation, chronic granulomatous disease (CGD), human immunodeficiency virus (HIV/AIDS), and autoimmune disorder accounted for 61.9% (n = 88).

**TABLE 1:**
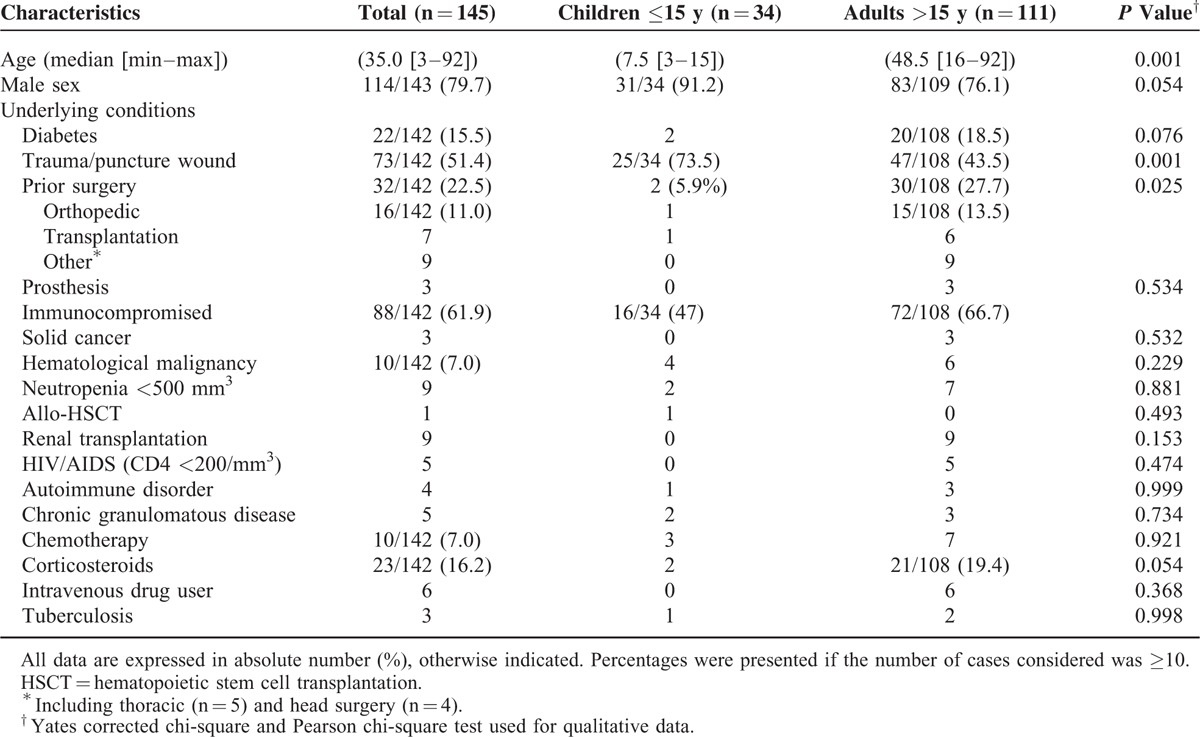
Demographic Characteristic of Non-*Aspergillus* Filamentous Fungi Osteoarticular Infections Reported in Adults and Children Between 1970 and 2003

### Etiologies and Mechanism of Infection

Overall, the most common groups of fungi involved in non-*Aspergillus* filamentous fungal osteoarticular infections were hyalohyphomycetes (n = 97), dematiaceous moulds (n = 25), and Mucorales (n = 23). The most common species involved in osteoarticular infections were *S. apiospermum, L. prolificans*, and *S. boydii*, followed by *Fusarium solani*. Most patients were infected with 1 fungal species. The fungal species was not specified in 30 cases (Table 2). The distribution of non-hyalohyphomycete species depended on the patient's age. Mucormycosis and the majority of phaeohyphomycosis cases were responsible for osteomyelitis in adults. *Chrysosporium zonatum* (hyalohyphomycosis) and *Myceliophthora thermophila* (phaeohyphomycosis) were isolated only in children. Hyalohyphomycoses were more frequently localized in lower limbs and axial skeleton compared with other groups of fungi.

**TABLE 2:**
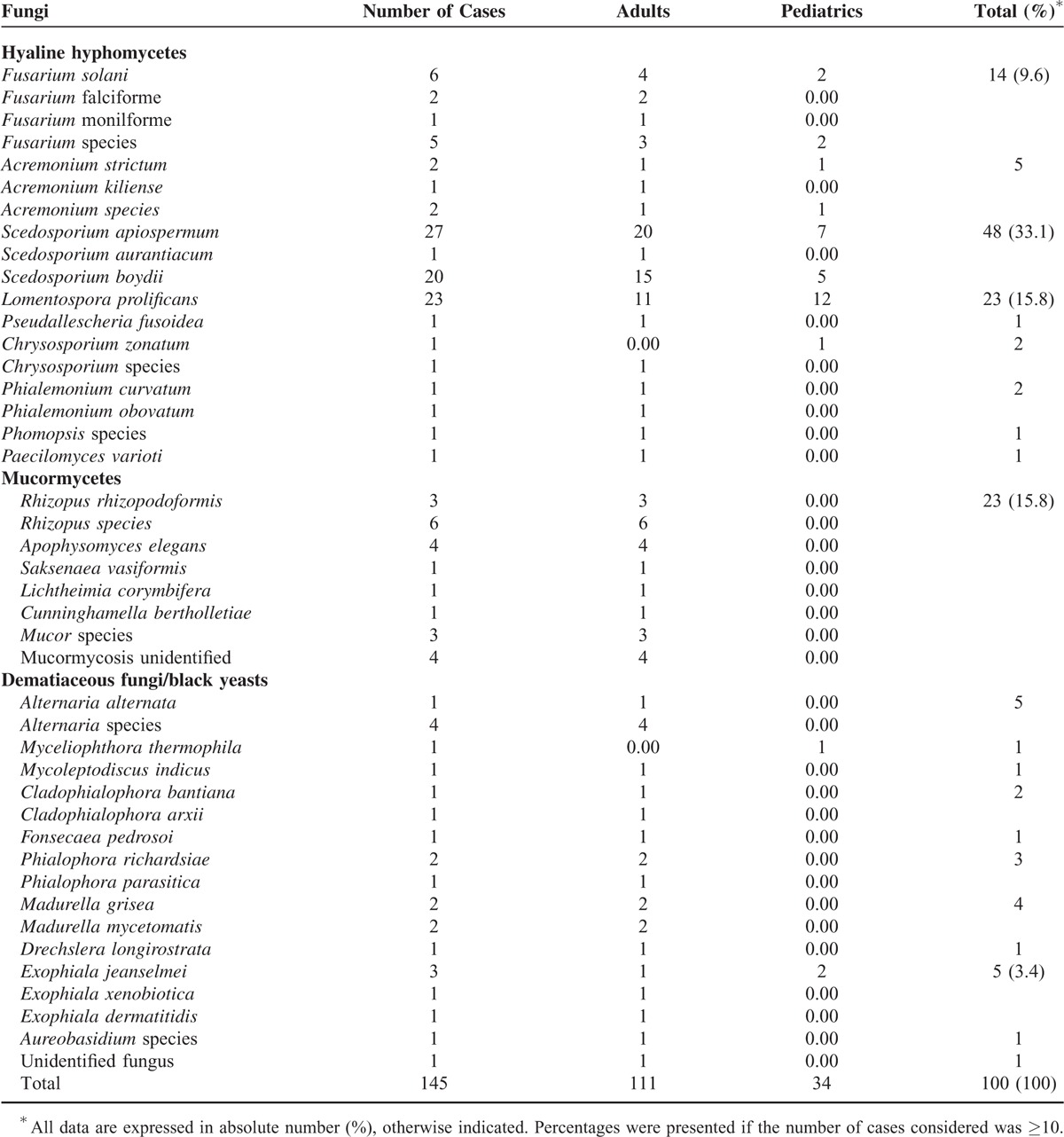
Distribution of Filamentous Fungi Causing Osteoarticular Infections in Adult and Pediatric Patients

The main mechanism of infection was direct inoculation 79 (54.5%), especially in children (n = 27/34, 79.4%), followed by hematogenous dissemination (24.8%), and contiguous spread (20.7%). Infection by direct inoculation in children was significantly higher than that observed in adults (79.4% vs 46.8%; *P* = 0.0007). Contiguous types of infections were significantly higher in adult patients (*P* = 0.0007). No significant difference was found in hematogenous dissemination between adults and children (*P* > 0.5).

**FIGURE 3:**
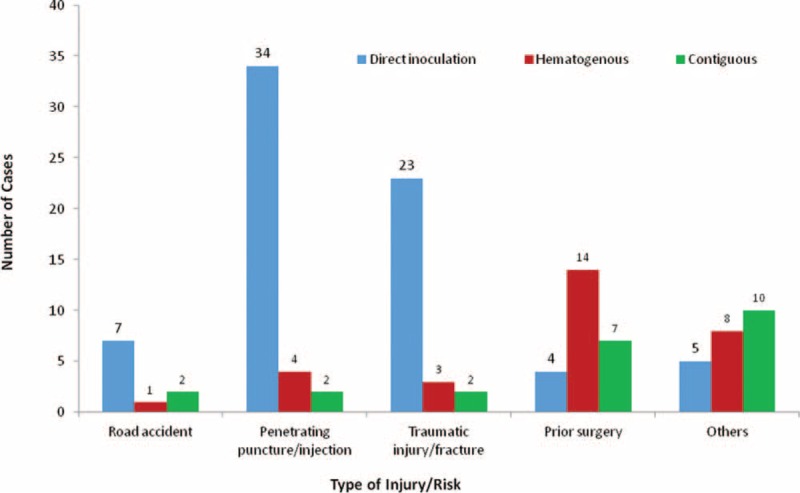
Significant association between injury and types of osteoarticular infections due to non-*Aspergillus* filamentous fungi (Overall *P* < 0.001).

Among the underlying conditions in immunocompetent patients were road accidents with fracture and puncture of the knee or penetrating wounds. Figure 3 shows a significant association between injury and types of apparent infections (*P* < 0.001). The highest risk surgical procedure was orthopedic followed by transplantation (Table 1). Prior surgery was significantly more frequent in adults (27.7% vs 5.9%; *P* = 0.025).

### Diagnostic Procedures and Cocultured Bacteria

Median time to diagnosis from onset of symptoms was 90 (7–1825) days. Among 139 diagnostic procedures, diagnosis of fungal osteomyelitis was performed by open biopsy in 96 (69%), percutaneous biopsy in 31 (22.3%), arthroscopy in 7 (5%), and other surgical procedures in 5 (3.6%). From these specimens, fungi were detected by culture, direct microscopy, and histology. Bacteria were cocultured from the same specimen in 22 cases; documented bacteria included *Staphylococcus aureus* followed by Gram-negative organisms, including *Escherichia coli* and *Pseudomonas aeruginosa* (data not shown).

### Clinical and Laboratory Features

The most frequently reported clinical manifestations were local pain and tenderness (69.0%), local inflammatory signs (44.1%), and restricted movements (53.5%). Children presented with significantly more local inflammatory signs than adults, 76.4% vs 34.2% (*P* = 0.0001). Fever was reported in only 31.5% of patients, significantly more frequently in the pediatric patients than in adults (61.2% vs 22.2%; *P* = 0.001). Dissemination of the infection was documented in 21/145(14.4%) patients, including 12 (57.1%) immunocompromised patients. De novo fungal osteoarticular infections occurred in 93.1% (n = 135) of the patients (Table 3).

**TABLE 3:**
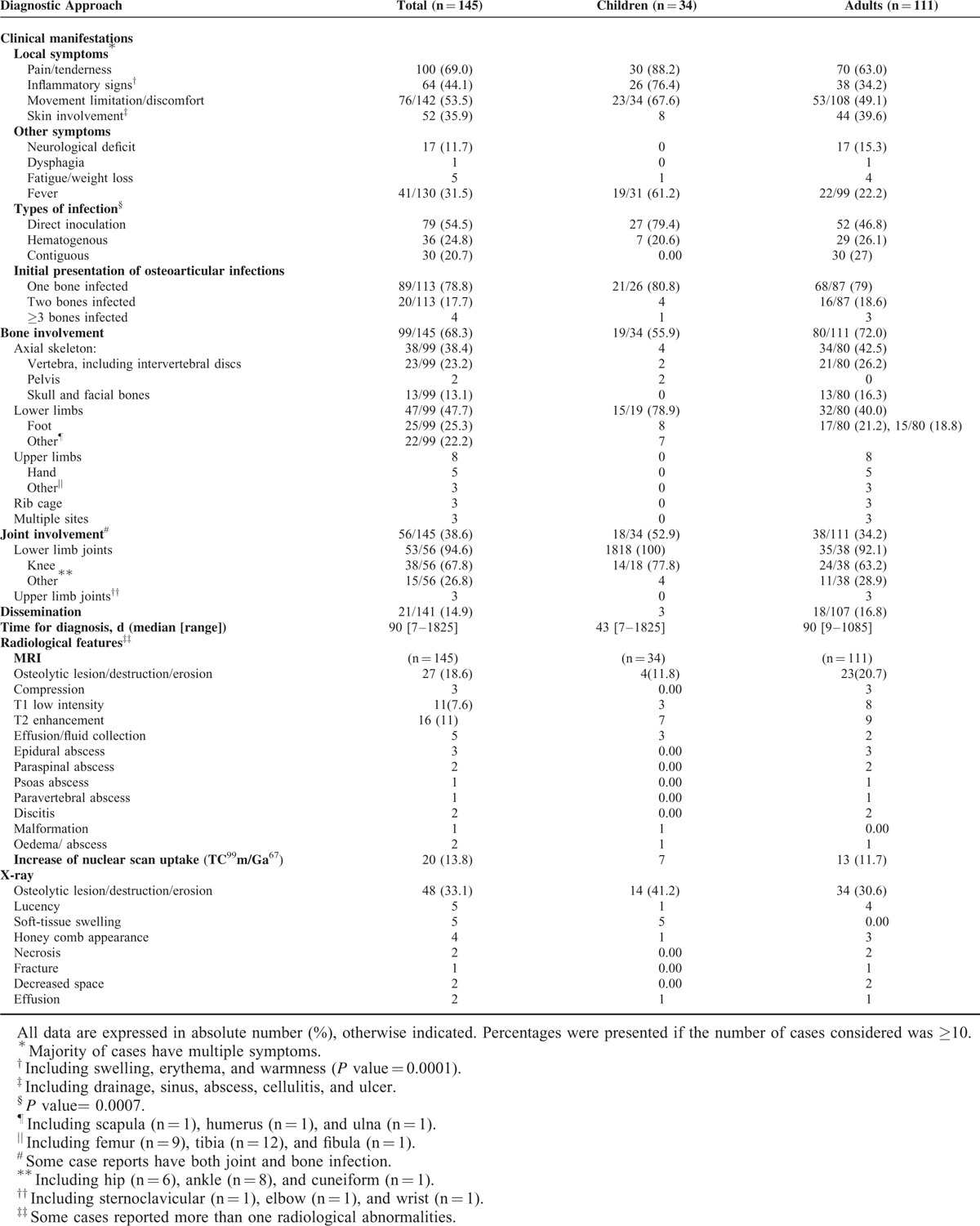
Clinical Characteristics and Anatomical Distribution of Osteoarticular Infections due to Non-*Aspergillus* Filamentous Fungi Reported in the Literature From 1970 to 2013

Among 99 cases of osteomyelitis caused by non-*Aspergillus* moulds, the most commonly involved sites were the lower limbs, particularly the foot (25.3%), vertebrae (23.2%), and the skull (13.1%). The lower limbs were more frequently involved in children than in adults (78.9% vs. 40.0%; *P* = 0.001). Vertebral osteomyelitis arose from hematogenous spread from pulmonary infections or occasionally by direct inoculation. Vertebral destruction/compression or cranial osteomyelitis led to neurological deficits in 8 and 9 cases, respectively. All such cases occurred in adult patients (n = 15, 10.3%). Among 56 cases of septic arthritis caused by non-*Aspergillus* filamentous fungi, the most common joint infected was the knee (67.8%) in both adults and children.

Elevated biomarkers of inflammation were detected in most tested patients. The median CRP value was 45 mg/dL (1.1–362), which was elevated to >5 mg/dL in 87.8% (n = 33) and to >1.0 mg/dL in 100% of tested patients. Significantly higher mean values of CRP were reported for pediatric patients than adult patients (110.2 ± 130.1 vs 46.7 ± 39.0; *P* = 0.034). An elevated WBC count was observed in 47.5% of the patients (n = 40), although the median value was 9850 cells/mm^3^ (1900–33,500).

### Diagnostic Imaging

The radiological abnormalities seen by different radiological techniques included osteolytic lesions, lucencies, vertebral compressions, abscesses, and increase of radionuclide uptake. Epidural, paraspinal, and psoas abscesses were detected only in adult patients. Magnetic resonance imaging (MRI) showed decreased signal intensity on T1-weighted images, as well as increased signal intensity on T2-wieghted images. Vertebral compression or decreased intervertebral space detected by plain radiographs was most commonly encountered in adult patients (Table 3).

### Treatment and Outcome

Surgical intervention and/or medical therapy was reported in 137 (94.5%) patients (Table 4). The majority of patients (69.3%) were treated with antifungal agents and surgery, 25.5% with antifungal agents only, and 5% (n = 7) with surgical treatment only. Amphotericin B (AmB) and voriconazole (VRC) were the 2 most commonly used antifungal agents in both children and adults. Combinations of antifungal therapy were reported in 25.4% of patients.

**TABLE 4:**
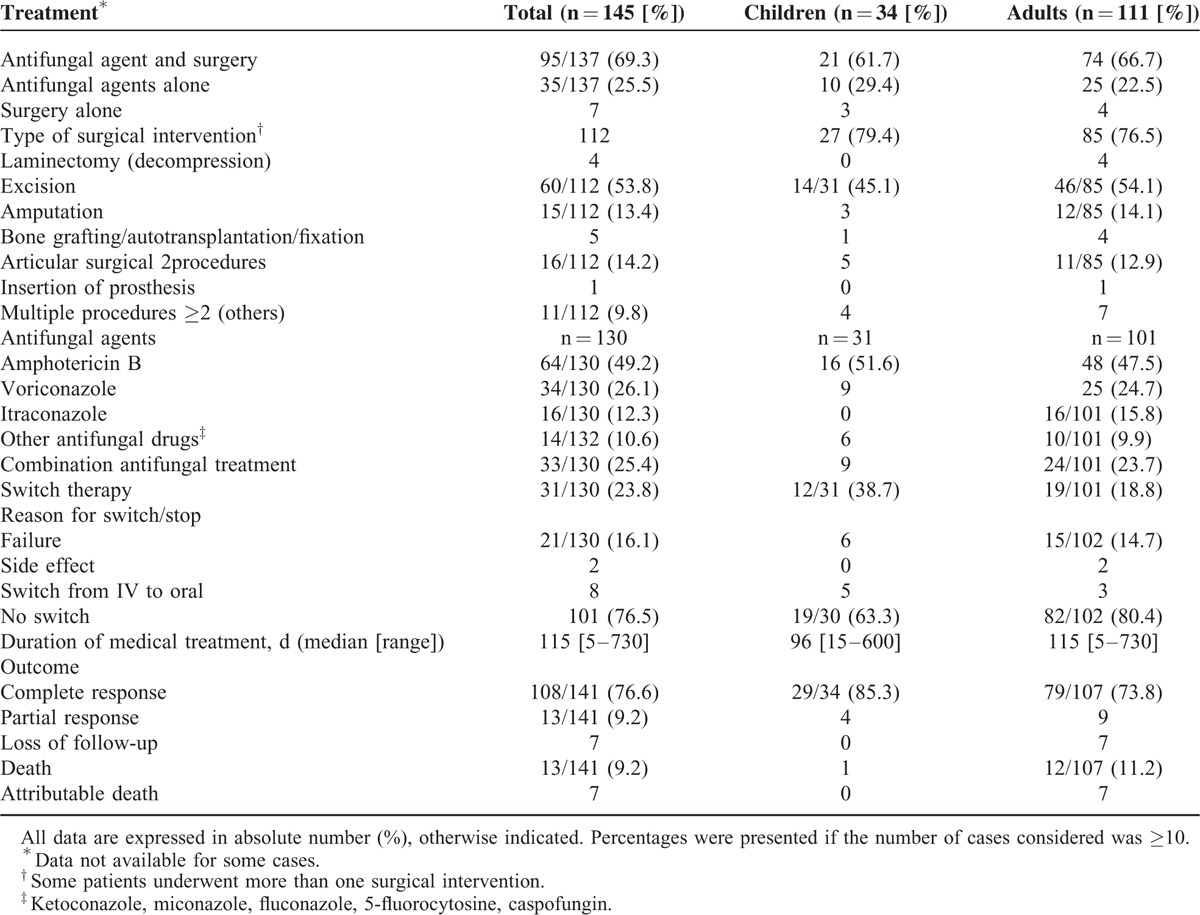
Treatment Strategy and Outcome of Non-*Aspergillus* Fungal Osteoarticular Infections in Pediatric and Adult Patients Reported in the Literature From 1970 to 2013

Hyalohyphomycosis was treated with AmB and VRC in 36 and 30 cases, respectively; overall responses included 3 deaths. All cases of mucormycosis were treated with AmB; treatment failure leading to death was noticed in 3 cases. Patients with phaeohyphomycosis were treated with AmB (n = 8), itraconazole (IT) (n = 9), and VRC (n = 3); 1 attributable death due to *Exophiala jeanselmei* was reported with AmB. A switch therapy was observed in 31 (23.5%) patients mainly due to treatment failure (15.9%). Switch therapy includes VRC, IT, posaconazole (POS) + terbinafine, or VRC + terbinafine.

Excision was the most common surgical intervention (52.6%), followed by articular surgical procedures (14%), amputation (13.1), and bone grafting. Regional infusion of antifungal agents was performed in 9 patients.

Median duration of medical treatment was 115 (5–730) days (Table 4). An overall response rate of 85.8% was achieved in the treatment of 141 non-*Aspergillus* mould osteomyelitis. complete response was reported in 108 (76%) patients, partial response in 13 (9.2%), and loss of follow-up in 7. Survival was 90.8% and overall mortality rate was 9.2% (n = 13/141). Irrespective of antifungal treatments, death was attributed to fungal osteoarticular infections in 7 adult patients.

## DISCUSSION

Reviewing 145 cases of fungal osteomyelitis and arthritis caused by non-*Aspergillus* filamentous fungi over 43 years, we found 43 fungal species belonging to 25 fungal genera of opportunistic and uncommon pathogens in immunocompetent and immunocompromised patients. This study displayed that 38.1% of osteoarticular infections due to these fungi occurred in apparently immunocompetent patients. Among the non-*Aspergillus* moulds, hyalohyphomycetes are the major group reported to cause osteoarticular infections. Two main pathogenic mechanisms of infection were observed: the first one was a community-acquired infection by direct inoculation during trauma, and the second one was a healthcare-associated infection after surgical procedures. Osteoarticular infections due to non-*Aspergillus* filamentous fungi were most of the times de novo infections and the rate of secondary dissemination was low. Fever was not a good clinical sign of osteoarticular infection, but local pain and inflammatory signs should alert the clinicians. Despite a low mortality rate, these osteoarticular infections were difficult to treat, and led to sequelae, especially when axial skeleton was involved.

As with *Aspergillus* and *Candida* osteoarticular infections,^1–5^ there is a high male predominance with >3.9:1 male-to-female ratio, and there were significant differences between the pediatric (≤15 y) and adult (>15 y) populations. In particular, trauma and puncture wounds, as well as lower limbs infections, were significantly more frequent in pediatric patients. Although there was no significant difference in the number of immunocompromised patients, adults had less clinical signs such as fever, movement limitation, or local inflammatory signs than children. Corticosteroids, which were mostly used in adults, could have masked the clinical signs in part of the adult population due to their anti-inflammatory effects. Another explanation could be driven by the differences in fungal species between children and adults. Hyalohyphomycosis predominated in children, whereas phaeohyphomycosis and mucormycosis were uncommon or absent in pediatric patients. Vertebral osteomyelitis occurred mainly in adults and was associated with neurological deficitsas in *Aspergillus* osteomyelitis.^1^

Biological markers may be useful in diagnosis and follow-up of some osteoarticular mycoses. Our findings revealed that an elevated level of CRP may support the clinical findings for early diagnosis of non-*Aspergillus* mould osteoarticular infections, whereas WBC count was minimally elevated. Unlike *Candida* osteomyelitis,^5^ CRP also may be useful for therapeutic monitoring of osteoarticular infections caused by non-*Aspergillus* spp.^1^

Surgical debridement, irrigation, and drainage of joints, combined with antifungal therapy, is widely considered the standard treatment option of fungal osteomyelitis and joint infections.^143^It is worthwhile mentioning that the majority of patients with *Aspergillus* (67%) and *Candida* (48%) osteoarticular infections received combined antifungal therapy and surgery.^1,5^

The treatment strategy for bone and joint infections due to non-*Aspergillus* filamentous fungi in the present study combined surgical and antifungal therapy in most cases. Therapeutic success, however, also depended upon the etiologic agent, the severity of the disease, type and location of infected bone, and the choice of antifungal agent. For example, *S. apiospermum* is more susceptible to antifungal agents than is *L. prolificans*. *S. apiospermum*, the related species *L. prolificans*, and other hyalohyphomycetes constituted 48.9% of osteoarticular infections. Treatment of osteomyelitis due to *S. apiospermum* often relies on a combination of antifungal therapy, surgical procedures, and occasionally amputation, when a poor prognosis is expected.^41,59^ The most potent in vitro activity has been observed with VRC.^144,145^ VRC, which was initially introduced in year 2001, has been used with good clinical results against *S. apiospermum* infections in immunocompromised patients, mostly for pulmonary and soft-tissue infections,^146,147^ and was recommended in the therapeutic standard in the European guidelines.^148^ Moreover, this agent has proved to be effective in treating Acremonium osteomyelitis,^22^and it has been shown to be effective in the treatment of other cases of *S. apiospermum* osteomyelitis where surgical amputation was avoided.^32^

As the antifungal susceptibility profile for *L. prolificans* showed higher resistance than *S. apiospermum*,^144,145^ identification of the fungus (from bone specimen) to the species level is important for the management of osteoarticular infections. The emergence of *Scedosporium aurantiacum*^84^and another related fungus, *Pseudallescheria fusoidea*,^37^ as new etiological agents of osteomyelitis, indicates that this group is an emerging cause of serious invasive diseases of bone in both immunocompetent and immunocompromised patients, and should be considered in the differential diagnosis of fungal osteomyelitis. In such cases, negative culture of synovial biopsy should be treated with a great deal of care, and a proper specimen based on bone biopsy is highly recommended. In addition, *Scedosporium* and *Fusarium* spp. are morphologically similar in histopathology sections.^149^ However, identification to species level is important to plan treatment strategy, as susceptibility to antifungal drugs is not similar.^144,145,150^

Osteoarticular mucormycosis constitutes a serious diagnostic and therapeutic challenge. Among 23 cases of osteomyelitis due to mucormycosis, all were treated with AmB and 3 had an unfavorable outcome. Osteoarticular mucormycosis is a highly destructive infection with poor prognosis, if not diagnosed early.^151^

*Fusarium* is highly refractory to treatment by antifungal agents with partial response and treated with either AmB^17^ or VRC.^14^ Susceptibility to antifungal agents is species specific.^152^ Koehler et al,^7^ based on combined treatment with VRC and AmB, suggested a management algorithm for treatment of *Fusarium* osteomyelitis.

The treatment option of these groups of fungi is based on combination of surgery and antifungal therapy in adults and pediatrics. It is interesting to note a considerably higher proportion of patients receiving the combination of surgery and antifungal therapy with a good survival rate (90.8%). Our findings also are consistent with case series by Koehler et al,^7^ who reported 61 cases of non-*Aspergillus* bone and joint infections, with a survival rate of 88%.

Unlike *Candida* and *Aspergillus* osteoarticular infections, which were reported as a result of hematogenous spread,^1,5^ direct inoculation is the cause of infection for the majority of non-*Aspergillus* filamentous fungal species reported herein. Patients with trauma, wounds, and punctures in this study comprised 51.4% of all cases of fungal osteoarticular infections. It constitutes the key risk factor of underlying conditions for development of knee arthritis and lower-limb osteomyelitis. Fungi rarely cause disease in healthy immunocompetent hosts. Disease results when fungi accidentally penetrate host barriers or when immunologic defects or other debilitating conditions exist, which favor fungal entry and infection. The etiologic agents gain entrance through transcutaneous puncture wounds, usually by a thorn or a splinter, or other kinds of trauma, such as road accident fractures, can be identified as the portal of entry of the fungus.^153^ Fungal spores may gain entrance to the host's soft tissue via plant fragments, or by injection with foreign body; the topography of the lesions on lower limbs, that is, feet and legs is typical presentation of direct inoculation particularly in pediatric patients of this study. A primary lesion can evolve to polymorphic skin lesions, including tenderness, swollen, erythema, or drainage sinus/abscess, which slowly enlarges and progress to osteomyelitis or arthritis.

This study identified previously unreported distinctions in the clinical manifestations, etiology, pathogenesis, and laboratory features between pediatric and adult patients with non-*Aspergillus* mould osteoarticular infections. Children presented with significantly more local signs than adults. Fever was present in twice the number of children than in adults. Epidural, paraspinal, and psoas abscesses were detected only in adult cases. As a preceding event, trauma and puncture wounds were nearly twice more frequent in children, whereas prior surgery was found more than 4 times more often in adults. Whereas lower-limb osteomyelitis and knee arthritis predominantly occurred in children, vertebral and craniofacial osteomyelitis with neurological deficits were common in adults. Infection by direct inoculation was a more frequent mechanism of infection in children than in adults. Finally, osteoarticular mucormycosis and phaeohyphomycosis predominated in adults, possibly as a reflection of a greater frequency of diabetes mellitus and immune impairment.

This study has several limitations. It is retrospective and thus will not capture all cases. There is the potential for publication bias, suggesting that cases that may have more favorable outcome are more likely to be published than those with poor outcome. Cognizant of these limitations, we believe that this study is the largest compilation and most detailed analysis of osteoarticular infections caused by medically important non-*Aspergillus* filamentous fungi. Moreover, due to the scarcity of data on these uncommon filamentous fungi causing osteoarticular infections, we believe that the case reports included in this study are likely representative enough of other cases. The data analysis of this study was based on detailed parameters of each single case to permit a high degree of analysis of several variables. Whereas an early study of literature reviewing 61 case series was highly informative in respect to detailed etiology and treatment, it lacked the numerical data for analysis of detailed clinical parameters, laboratory markers, as well as reporting only ten pediatric (≤15 y) patients.^7^

Non-*Aspergillus* osteoarticular mycoses occur most frequently in children after injury and in adults after surgery. These fungi can cause a serious illness and more virulent in individuals with impaired immune systems. Accurate early diagnosis and long-course therapy (median 6 mo) with a combined medical-surgical approach may result in favorable outcome.
